# Association of Serum GDF-15 with Bone and Muscle Parameters in Community-Dwelling Individuals

**DOI:** 10.3389/fmed.2026.1920342

**Published:** 2026-10-09

**Authors:** Nicia I. Profili, Sokratis Makrogiannis, Roberto Castelli, Maria Benelli, Valeria Orrù, Michele Marongiu, Edoardo Fiorillo, Francesco Cucca, Alessandro P. Delitala

**Affiliations:** 1 Department of Medicine, Surgery, and Pharmacy, University of Sassari, Sassari, Italy; 2 Division of Physics, Engineering, Mathematics, and Computer Science, Delaware State University, Dover, DE, United States; 3 Institute for Genetic and Biomedical Research, National Research Council, Monserrato, Italy; 4 Department of Biomedical Science, University of Sassari, Sassari, Italy

**Keywords:** aging, bone mineral density, GDF-15, pQCT, sarcopenia

## Abstract

**Introduction:**

Muscle mass and bone mineral density decrease with aging, although following different patterns among males and females. Growth differentiation factor 15 (GDF-15) is a stress-induced cytokine and has emerged as a key biomarker for inflammation, tissue injury, and metabolic stress. We tested the association between GDF-15 and specific bone and muscle parameters in a large cohort of community-dwelling subjects.

**Methods:**

The SardiNIA survey included 4,842 subjects (2,795 females). All underwent a detailed medical visit and laboratory testing. Peripheral quantitative computed tomography at the tibia was performed to evaluate volumetric bone mineral density and bone microstructure as well as muscle mass.

**Results:**

Multivariate regression analysis showed that GDF-15 was negatively associated with cortical bone density (Beta −0.042, *p* < 0.001) in females. In males, total bone density and trabecular bone density at 4% site and cortical bone area at 38% site were negatively associated with GDF-15 levels (respectively, Beta −0.016, *p* = 0.027, Beta −0.014, *p* = 0.039, and Beta −0.022, *p* = 0.002). Multivariate regression analysis for muscle parameters showed that GDF-15 was negatively associated with muscle density (Beta −0.002 in females and males, *p* < 0.001 for all) and muscle area (Beta −0.338, *p* = 0.003, Beta −0.744, *p* < 0.001).

**Conclusions:**

Higher circulating GDF-15 levels were independently associated with lower volumetricBMD and poorer muscle parameters in both women and men. These findings support the potential role of GDF-15 as a biomarker of age-related musculoskeletal decline, although longitudinal studies are needed to determine whether GDF-15 contributes to the development of these changes or simply reflects the underlying biological processes.

## 1 Introduction

Osteoporosis is a chronic bone disease diagnosed when bone mineral density (BMD), assessed by dual-energy x-ray absorptiometry (DXA), is 2.5 standard deviations below the average value for young healthy women, whereas osteopenia is diagnosed when BMD ranges between −1.0 and −2.5 standard deviations. Osteoporosis markedly increases the risk of fractures, which, in turn, are associated with increased morbidity and mortality (1). Although DEXA is currently the reference method for measuring areal BMD and is widely used for the diagnosis of osteoporosis, it provides limited information on bone geometry, cortical and trabecular compartments, and muscle characteristics. Peripheral quantitative computed tomography (pQCT) enables three-dimensional assessment of volumetric BMD, bone geometry, and muscle cross-sectional area and density, allowing a more comprehensive evaluation of musculoskeletal health and age-related tissue changes.

Bone tissue mineralization is critical in the development of osteoporosis. Genetic predisposition, estrogen or androgen deficiency, mechanical loading, and lifestyle factors are the main regulators of bone health (2). Previous evidence has shown that trabecular and cortical bone exhibit different patterns of age-related changes. Indeed, although both compartments undergo a progressive age-related decline, the rate of decline is steeper in women than in men. Both sexes also show an increase in bone cross-sectional area, probably as a compensatory adaptation to maintain adequate bone mechanical competence. However, this compensatory mechanism appears to be more efficient in males (3). Lifestyle plays a pivotal role in bone health, as demonstrated by substantial previous evidence. In particular, physical exercise reduces fracture risk and attenuates the age-associated decline in BMD. Sarcopenia is another common condition in the aging population and is characterized by a decline in skeletal muscle mass and function. Sarcopenia and osteoporosis share several risk factors, and several studies have demonstrated their association. Indeed, a recent meta-analysis showed that sarcopenia increases the risk of developing osteoporosis (4). Osteoporosis and sarcopenia are multifactorial age-related disorders that share several biological mechanisms, including chronic low-grade inflammation (“inflammaging”), mitochondrial dysfunction, oxidative stress, hormonal dysregulation, and impaired regenerative capacity (5–7). These mechanisms contribute to reduced osteoblast activity, increased osteoclastogenesis, loss of muscle mass, and deterioration of muscle quality. Growing evidence indicates that bone and skeletal muscle function as an integrated musculoskeletal unit through endocrine and paracrine signaling pathways involving myokines, osteokines, and inflammatory mediators. Therefore, biomarkers reflecting these shared biological pathways may provide valuable insights into the development of osteosarcopenia.

Growth differentiation factor-15 (GDF-15) belongs to the transforming growth factor-β (TGF-β) superfamily and is involved in several metabolic pathways. Previous studies have reported that variations in circulating GDF-15 levels are associated with aging, mitochondrial disorders, energy imbalance, thyroid diseases, as well as wasting and cachexia (8–10).

Evidence regarding the association between GDF-15 and sarcopenia is robust. Activation of the GDF-15 receptor at the hindbrain level may contribute to sarcopenia and weight loss (11, 12) and circulating GDF-15 levels have been positively correlated with sarcopenia (13). Previous studies have also demonstrated that GDF-15 concentrations are higher in older sarcopenic patients admitted to hospital (14). Furthermore, circulating GDF-15 levels have been associated with reduced isometric quadriceps strength (15).

The association between bone metabolism and GDF-15 remains unclear. Previous studies have shown that GDF-15 is produced by osteocytes (16) and promotes osteoclast differentiation in mice, thereby contributing to bone loss. Moreover, GDF-15 expression is modulated by vitamin K in human osteoblastic cells (17). Clinical studies in patients with specific diseases supported the potential role of GDF-15 as a biomarker of osteoporosis (18), and a large-scale meta-analysis revealed that increased GDF-15 levels are associated with a higher risk of hip fractures (19). Although previous studies have reported associations between circulating GDF-15 and sarcopenia or reduced muscle mass, most were conducted in selected clinical populations and focused primarily on muscle-related outcomes. Whether GDF-15 is associated with both bone and muscle parameters assessed by pQCT in community-dwelling adults remains largely unexplored.

## 2 Methods

SardiNIA is an ongoing longitudinal cohort study investigating thousands of genetic variants associated with aging (20). In 2001, all the residents of four towns in the Lanusei valley (Lanusei, Arzana, Ilbono, and Elini) were invited to participate in the study. Among the 9,841 individuals eligible for recruitment, 6,148 participants were recruited, most of whom were native to the region. The survey is structured in waves of visits, each of which lasts about 3–4 years. Five waves have been completed, and the sixth is currently ongoing.

In every wave, participants had a clinical visit performed by trained staff. The visit included blood sample collection, assessment of specific cardiovascular parameters (blood pressure, non-invasive measures of arterial structure and function, and specific geriatric tests). The analysis of this study is based on data from the third wave of visits.

All participants provided written informed consent. The study protocol was approved by the Sardinian Regional Ethics Committee (protocol no. 2171/CE, approved on May 19, 2015).

### 2.1 Biochemical assays

Blood samples were drawn between 7 and 8 a.m. after overnight fasting and stored at −80 °C until use. Total cholesterol and triglycerides were assessed by an enzymatic method (Abbott Laboratories ABA-200 ATC Biochromatic Analyzer, Irving, TX, USA). High-density lipoprotein (HDL) cholesterol was quantified by dextran sulphate–magnesium precipitation. Low-density lipoprotein (LDL) cholesterol was calculated using the Friedewald formula: LDL cholesterol = total cholesterol−(HDL cholesterol + (triglycerides/5).

Serum GDF-15 level was assessed by ELISA (DY957 R&D Systems). Optical density was measured at 450 nm with wavelength correction at 570 nm. All measurements fell within the range of the standard curve (7.8–1,000 pg/mL). Measurements were performed on a single lot of reagents to avoid batch effects. All measurements were performed in duplicate, and the average of the two replicate values was used for the subsequent analyses.

### 2.2 Bone and muscle assessment

Bone geometry was assessed on the non-dominant tibia by Peripheral Quantitative Computed Tomography (pQCT) scanner XCT2000 (Stratec Medizintechnik GmBH, Pforzheim, Germany). Scan acquisition parameters were as follows: voxel size of 0.5 × 0.5 mm, slice thickness of 2 mm, and the scan speed was 30 mm/s. Measurements were obtained at 4% and 38% of the limb length proximal to the distal endplate of the tibia. Structure of Tissue Identification And Quantification (TIDAQ) software was used to compute bone and muscle variables (21).

### 2.3 Covariates

BMI was calculated as body weight in kilograms divided by height squared (m^2^). Subjects were classified as normal weight (18.5–24.9 kg/m^2^), overweight (25.0–29.9 kg/m^2^), or obese (≥ 30.0 kg/m^2^). For the purpose of this study, comparison across categories of BMI did not include underweight subjects.

### 2.4 Statistical analysis

Normality was assessed using the Shapiro–Wilk test. Because none of the continuous variables were normally distributed, data are presented as median and interquartile range. Comparisons between two independent groups were performed using the Wilcoxon rank-sum test, whereas comparisons among more than two groups were performed using the Kruskal–Wallis test. Correlations between continuous variables were assessed using Spearman's rank correlation coefficient.

Because serum GDF-15 concentrations were right-skewed, log-transformed values were used in correlation and regression analyses. Separate sex-stratified multivariable linear regression models were fitted for each pQCT outcome using heteroscedasticity-robust (Huber–White) standard errors to account for heteroscedasticity. Model performance was evaluated using the adjusted R^2^, and multicollinearity was assessed using variance inflation factors (VIFs). Log-transformed GDF-15 was entered as the independent variable of interest, whereas age, waist circumference, BMI, and menopausal status (women only) were included as covariates. Regression diagnostics were performed before model interpretation. Linearity and multicollinearity were assessed. Regression coefficients are reported as beta coefficients with standard errors, 95% confidence intervals, and two-sided *p* values. Statistical significance was set at a two-sided *p* value <0.05. All analyses were performed using Stata version 18.0 (StataCorp, College Station, TX, USA).

## 3 Results

### 3.1 Baseline characteristics

The sample consisted of 4,842 participants (57.7% female), as shown in Table 1. Males were older (49 vs. 48 years, *p* < 0.05), had a higher waist and BMI (respectively, 93 vs. 82 cm and 27 vs. 24 Kg/m^2^, *p* < 0.001). Females had lower HbA1c level (5.4% vs. 5.3%, *p* < 0.001) and had higher levels of total cholesterol (215 vs. 212 mg/dL, *p* < 0.01) and HDL (60 vs. 49 mg/dL, *p* < 0.001) compared with males, which had higher triglycerides levels (111 vs. 89 mg/dL, *p* < 0.001). Total and trabecular bone density as well as total bone area at 4% level were higher in males as compared with female (283 vs. 239 mg/cm^3^, 225 vs. 189 mg/cm^3^, and 1,110 vs. 913 mm^2^, *p* < 0.001 for all). Similarly, cortical bone area at 38% level (370 vs. 275 mm^2^, *p* < 0.001), and muscle area (7387 vs. 5603 mm^2^, *p* < 0.001) were higher in males. Females had higher fat area (2,562 vs. 1,345 mm^2^, *p* < 0.001). LDL, GDF-15, cortical bone density at 38% level, and muscle density were comparable between males and females.

**Table 1:** Summary of characteristics of the sample.

	Female	Male	Total
*n*	2,795	2,047	4,842
Age, yrs	48 (37–61)	49 (37–64)*	49 (37–62)
Waist, cm	82 (75–90)	93 (86–100)***	87 (78–95)
BMI, Kg/m^2^	24 (21–28)	27 (24–29)***	26 (23–29)
HbA1c, %	5,3 (5–5.6)	5,4 (5–5.7)***	5,3 (5–5.6)
Total cholesterol, mg/dL	215 (190–242)**	212 (183–240)	214 (187–241)
LDL, mg/dL	133 (112–155)	135 (110–160)	133 (111–157)
HDL, mg/dL	60 (52–69)	49 (43–57)***	55 (47–65)
Triglycerides, mg/dL	89 (66–124)	111 (81–157)***	98 (72–138)
GDF-15, pg/mL	205 (158–279)	205 (156–291)	205 (157–282)
Total bone density 4%, mg/cm^3^	239 (214–264)	283 (255–315)***	256 (227–287)
Trabecular bone density 4%, mg/cm^3^	189 (168–212)	225 (200–253)***	204 (179–231)
Cortical bone density 38%, mg/cm^3^	1,066 (1,028–1,097)	1,066 (1,036–1,093)	1,066 (1,031–1,095)
Cortical bone area 38%, mm^2^	275 (253–296)	370 (341–400)***	307 (269–363)
Total bone area 4%, mm^2^	913 (837–996)	1,110 (1,014–1,209)***	989 (883–1,104)
Muscle density, mg/cm^2^	74 (73–76)	74 (72–76)	74 (72–76)
Muscle area, mm^2^	5,603 (5,091–6,159)	7,387 (6,705–8,137)***	6,237 (5,436–7,286)
Fat area, mm^2^	2,562 (2,013–3,234)	1,345 (967–1,793)***	2,020 (1,374–2,787)

BMI, body mass index; HbA1c, glycated hemoglobin; LDL, low density lipoprotein cholesterol; HDL, high density lipoprotein cholesterol, GDF-15, growth differentiation factor-15.

* *p* < 0.05.

** *p* < 0.01.

*** *p* < 0.001.

### 3.2 Bone-related outcomes

Figure 1 shows the variations of GDF-15 across BMI categories. Log transformed GDF-15 progressively increased from participants with normal weight to those with obesity, in both males and female (*p* < 0.001 for all).

**Figure 1:**
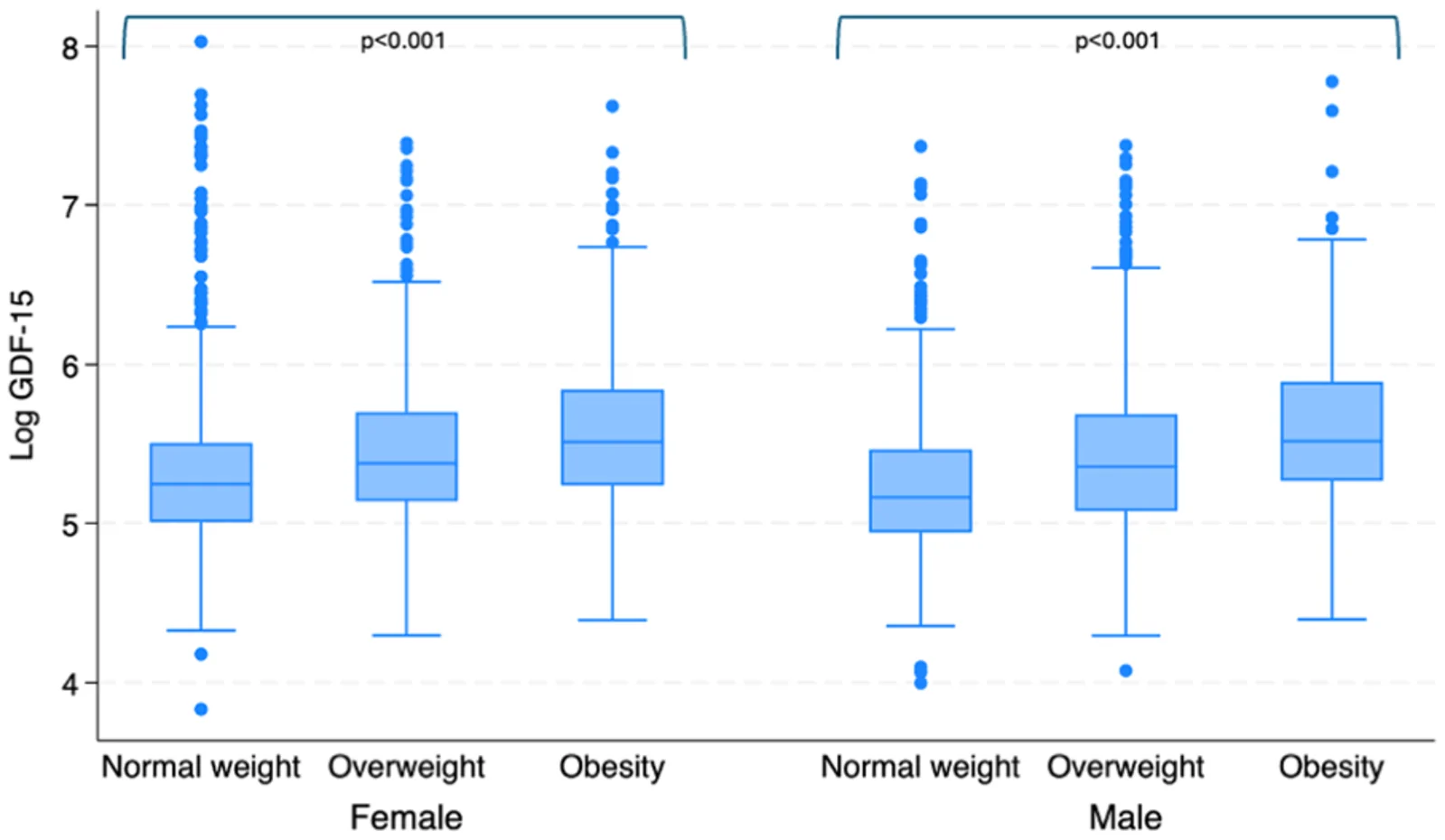
Variations of GDF-15 across BMI categories. Log GDF-15, logarithm of growth differentiation factor-15.

At univariate analysis in females (Figure 2), Log GDF-15 was negatively associated with total vBMD and trabecular vBMD at 4% level (rho −0.284, rho −0.210 *p* < 0.001), as well as with cortical vBMD at 38% level (rho −0.351, *p* < 0.001). On the contrary, total bone area at 4% level was positively associated with log GDF-15 (rho 0.201, *p* < 0.001), which was not significantly correlated to cortical bone area at 38% level.

**Figure 2:**
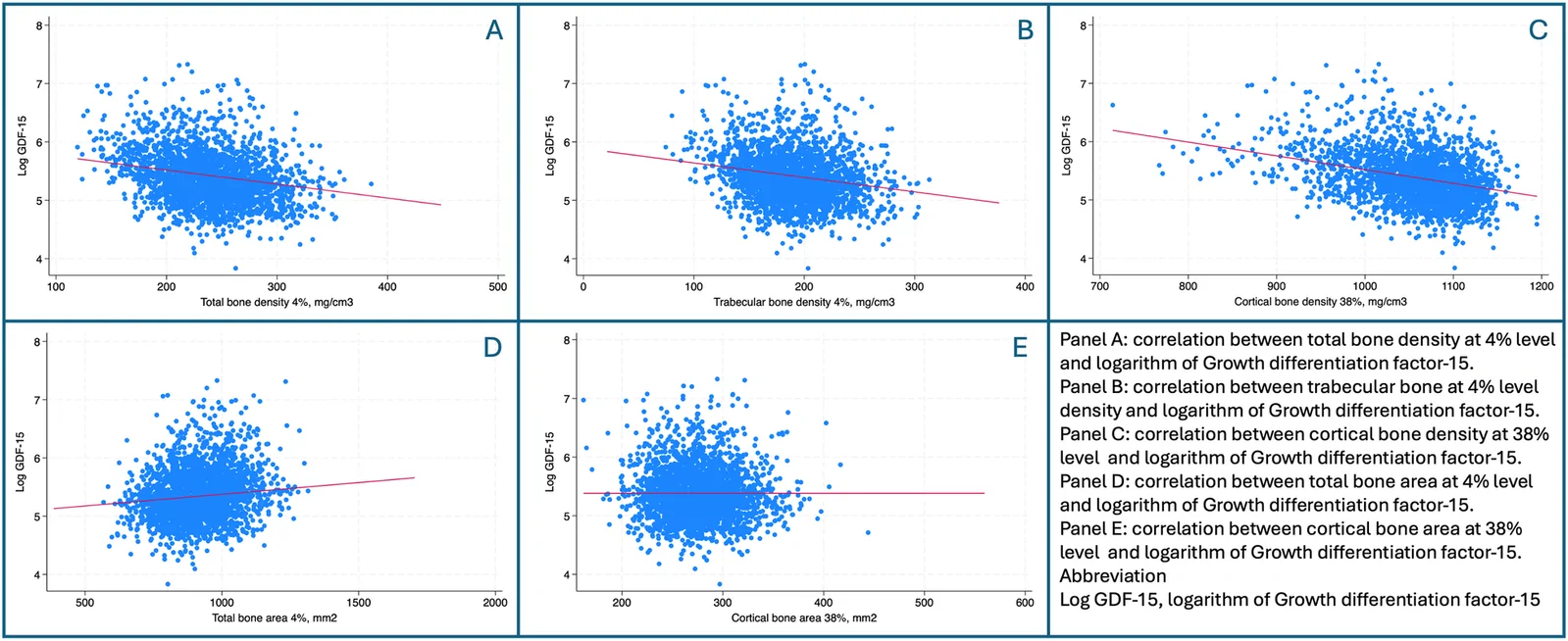
Correlation between log GDF-15 and bone pQCT parameters in females. **(A)** Correlation between total bone density at 4% level and logarithm of Growth differentiation factor-15. **(B)** Correlation between trabecular bone at 4% level density and logarithm of Growth differentiation factor-15. **(C)** Correlation between cortical bone density at 38% level and logarithm of Growth differentiation factor-15. **(D)** Correlation between total bone area at 4% level and logarithm of Growth differentiation factor-15. **(E)** Correlation between cortical bone area at 38% level and logarithm of Growth differentiation factor-15. Log GDF-15, logarithm of Growth differentiation factor-15.

Males showed a similar pattern to that observed in females, as reported in Figure 3. Log GDF-15 was negatively associated with total vBMD and trabecular vBMD at 4% level (rho −0.275, rho −0.275 *p* < 0.001), and cortical vBMD at 38% level (rho −0.101, *p* < 0.001), while total bone area at 4% level was positively associated with log GDF-15 (rho 0.123, *p* < 0.001). Log GDF-15 was not significantly correlated to cortical bone area at 38% level.

**Figure 3:**
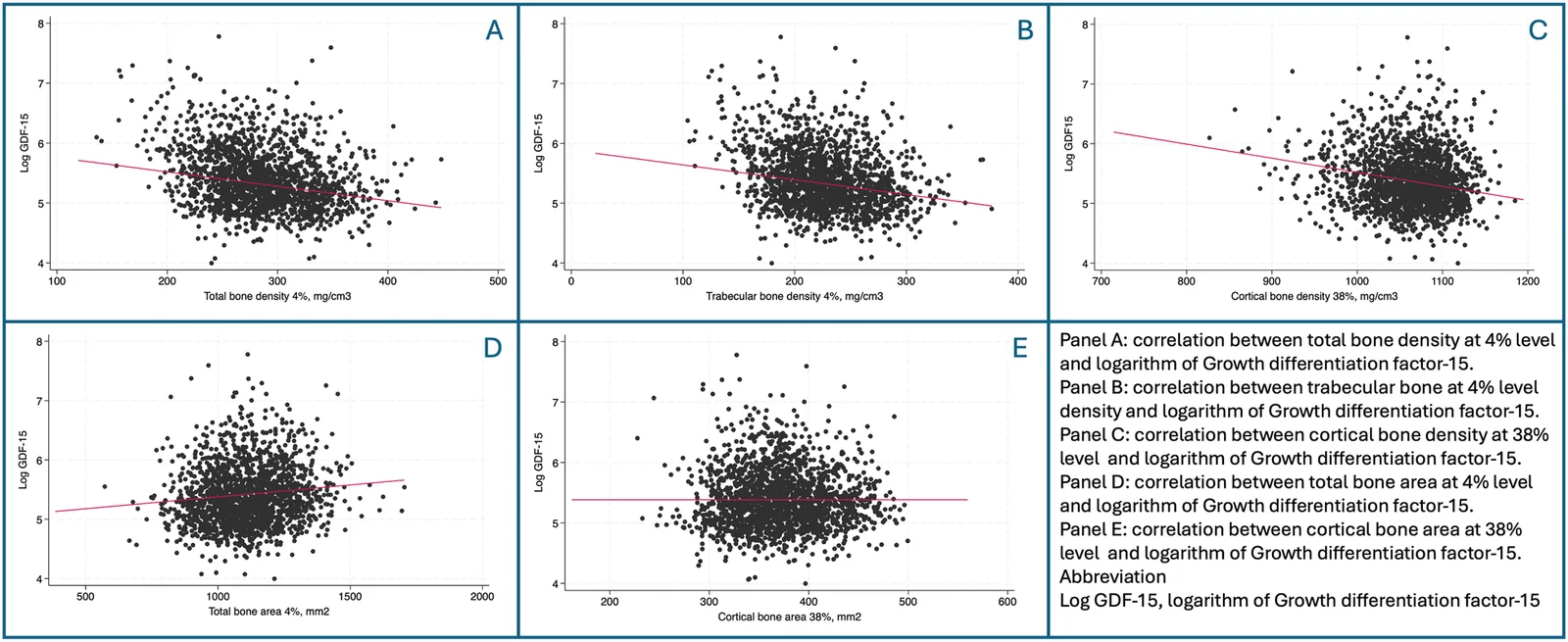
Correlation between log GDF-15 and bone pQCT parameters in males. **(A)** Correlation between total bone density at 4% level and logarithm of Growth differentiation factor-15. **(B)** Correlation between trabecular bone at 4% level density and logarithm of Growth differentiation factor-15. **(C)** Correlation between cortical bone density at 38% level and logarithm of Growth differentiation factor-15. **(D)** Correlation between total bone area at 4% level and logarithm of Growth differentiation factor-15. **(E)** Correlation between cortical bone area at 38% level and logarithm of Growth differentiation factor-15. Log GDF-15, logarithm of Growth differentiation factor-15.

### 3.3 Muscle related outcomes

As illustrated in Figures 4, 5, log GDF-15 was negatively associated with muscle density (rho −0.338 in females and rho −0.434 in males, *p* < 0.001 for all) and muscle area (rho −0.434 in females and −0.112 in males, *p* < 0.001 for all). Fat area was not associated with GDF-15 in both males and females.

**Figure 4:**
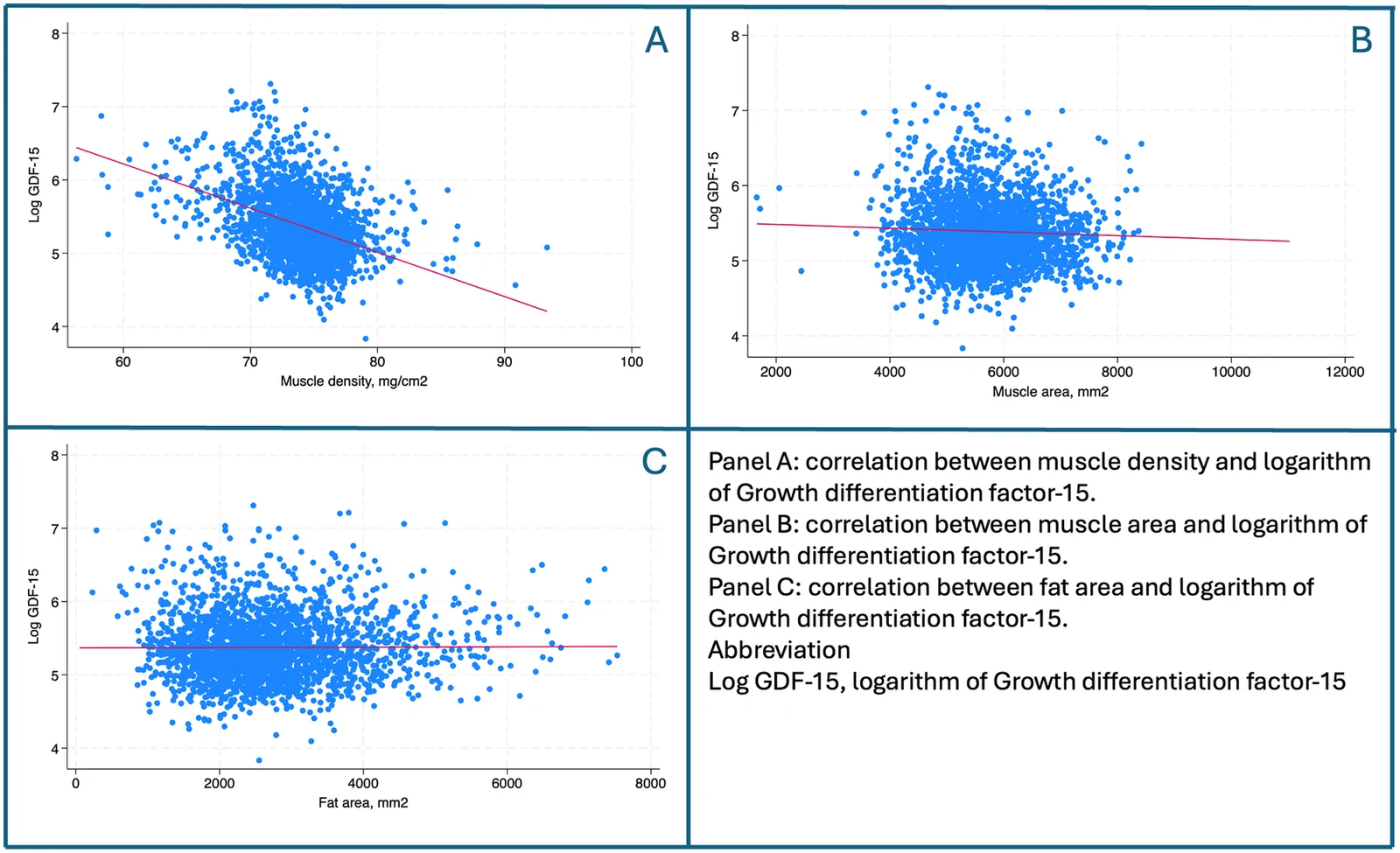
Correlation between log GDF-15 and muscle pQCT parameters in females. **(A)** Correlation between muscle density and logarithm of Growth differentiation factor-15. **(B)** Correlation between muscle area and logarithm of Growth differentiation factor-15. **(C)** Correlation between fat area and logarithm of Growth differentiation factor-15. Log GDF-15, logarithm of Growth differentiation factor-15.

**Figure 5:**
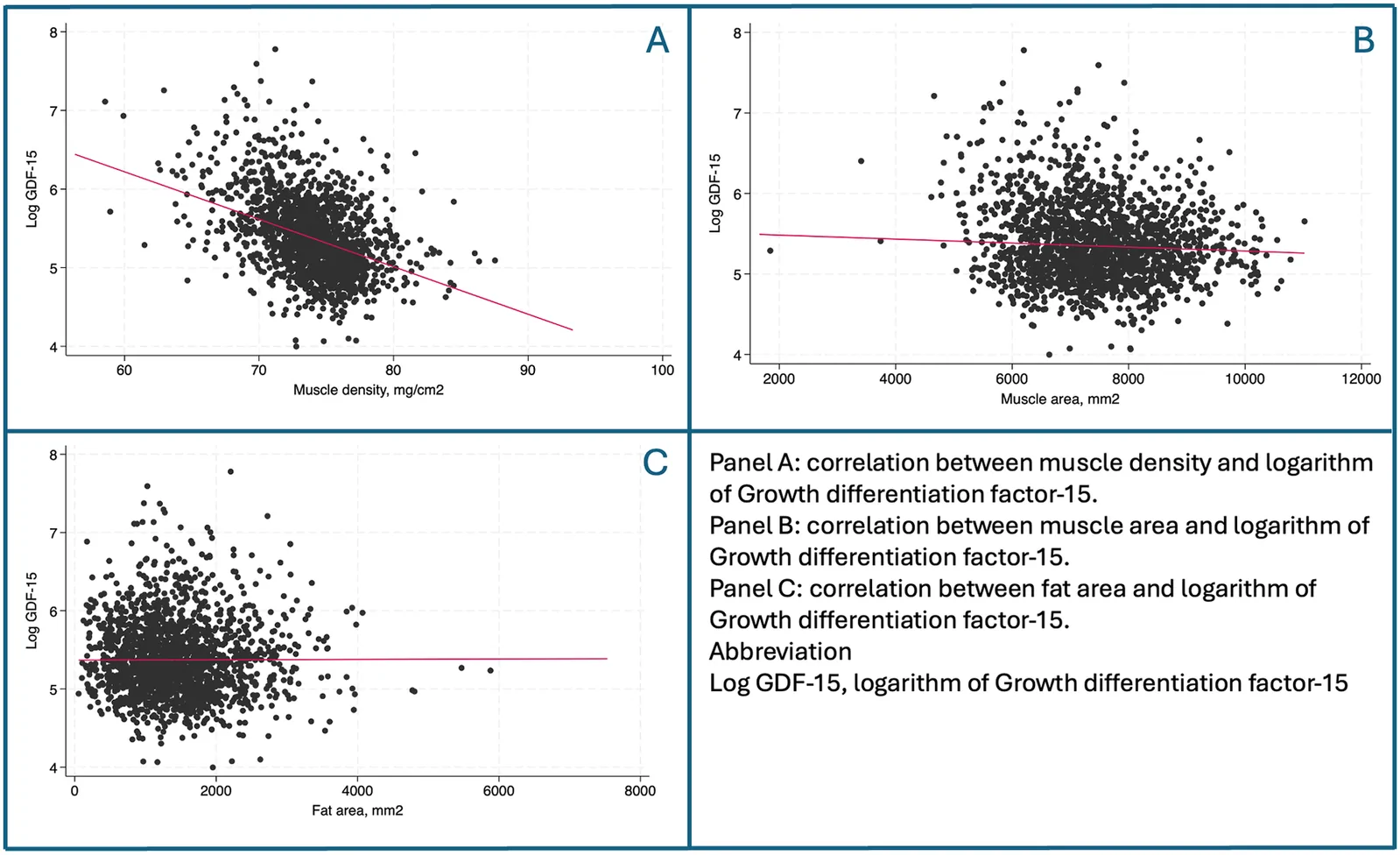
Correlation between log GDF-15 and muscle pQCT parameters in male. **(A)** Correlation between muscle density and logarithm of Growth differentiation factor-15. **(B)** Correlation between muscle area and logarithm of Growth differentiation factor-15. **(C)** Correlation between fat area and logarithm of Growth differentiation factor-15. Log GDF-15, logarithm of Growth differentiation factor-15.

### 3.4 Multivariable analyses

Table 2 lists the results of sex-stratified multivariable analyses of pQCT bone parameters. In female participants, GDF-15 was negatively associated with cortical vBMD (Beta −0.042, *p* < 0.001), whereas in males total vBMD and trabecular vBMD at 4% level and cortical bone area at 38% level were negatively associated with GDF-15 (respectively, Beta −0.016, *p* = 0.027, Beta −0.014, *p* = 0.039, and Beta −0.022, *p* = 0.002).

**Table 2:** Association between GDF-15 and bone pQCT parameters: results of multivariate regression analysis.

	Female					Male				
	B	t	s.e.	p	95% CI	B	t	s.e.	p	95% CI
Total bone density 4%										
Age	−1.304	−16.48	0.079	<0.001	−1.46; −1.15	−0.984	−11.61	0.075	<0.001	−1.15; −0.82
GDF-15	−0.002	−0.34	0.006	0.730	−0.01; 0.01	−0.016	−1.76	0.008	0.027	−0.03; −0.01
Waist	−0.041	−0.39	0.107	0.699	−0.25; 0.17	−0.595	−1.77	0.336	0.077	−1.25; −0.06
BMI	2.876	11.64	0.247	<0.001	2.39; 3.36	3.281	4.14	0.791	<0.001	1.72; 4.83
Menopause	−8.735	−3.84	2.273	<0.001	−13.19; −4.28	–	–	–	–	–
Trabecular bone density 4%										
Age	−1.131	−14.85	0.076	<0.001	−1.28; −0.98	−0.893	−12.16	0.073	<0.001	−1.04; −0.75
GDF-15	0.005	0.95	0.005	0.344	−0.01; 0.02	−0.014	−2.06	0.007	0.039	−0.03; −0.01
Waist	0.033	0.34	0.100	0.736	−0.16; 0.23	−0.351	−1.24	0.283	0.215	−0.90; −0.20
BMI	2.324	9.68	0.240	<0.001	1.85; 2.79	2.330	3.50	0.665	<0.001	1.03; 3.63
Menopause	−1.623	−0.76	2.137	0.447	−5.81; 2.56	–	–	–	–	–
Cortical bone density 38%										
Age	−1.084	−8.14	0.133	<0.001	−1.35; −0.82	−0.291	−3.48	0.083	<0.001	−0.46; −0.13
GDF-15	−0.042	−4.43	0.009	<0.001	−0.06; −0.02	−0.001	−0.06	0.007	0.954	−0.01; 0.01
Waist	−0.328	−2.28	0.143	0.022	−0.61; −0.05	−0.060	0.28	0.213	0.778	−0.36; 0.47
BMI	0.888	2.57	0.344	0.010	0.21; 1.56	−1.076	−2.02	0.533	0.044	−2.12; −0.03
Menopause	−26.602	−7.87	3.379	<0.001	−33.23; −19.98	–	–	–	–	–
Cortical bone area 38%										
Age	−0.381	−4.76	0.080	<0.001	−0.54; −0.22	−0.311	−3.91	0.079	<0.001	−0.47; −0.16
GDF-15	0.003	0.45	0.006	0.655	−0.01; 0.01	−0.022	−3.07	0.007	0.002	−0.04; −0.01
Waist	0.539	5.61	0.096	<0.001	0.35; 0.73	0.428	2.39	0.179	0.017	0.08; 0.78
BMI	1.917	7.96	0.240	0.002	1.44; 2.39	3.430	7.36	0.466	<0.001	2.52; 4.34
Menopause	−6.710	−3.11	2.216	<0.001	−10.94; −2.48	–	–	–	–	–
Total bone area 4%										
Age	1.113	4.00	0.278	<0.001	0.57; 1.66	0.866	3.29	0.269	0.001	0.34; 1.39
GDF-15	0.024	1.18	0.020	0.237	−0.15; 0.06	−0.025	−1.08	0.023	0.282	−0.07; 0.02
Waist	1.761	4.59	0.383	<0.001	1.01; 2.51	3.776	5.69	0.978	<0.001	1.86; 5.69
BMI	−2.200	−2.55	0.861	0.011	−3.89; −0.52	−5.528	−2.23	2.360	0.019	−10.16; −0.90
Menopause	31.409	3.97	7.913	<0.001	15.89; 46.93	–	–	–	–	–

B, beta coefficients; t, t-value; s.e. standard error; 95% CI, 95% confidence interval.

As reported in Table 3, after adjusting for age, waist, BMI, and menopause (in female) GDF-15 was negatively associated to muscle density (Beta −0.002 in both female and males, *p* < 0.001 for all) and to muscle area (Beta −0.338, *p* = 0.003 in female, Beta −0.744, *p* < 0.001 in male).

**Table 3:** Association between GDF-15 and muscle pQCT parameters: results of multivariate regression analysis.

	Female					Male				
	B	t	s.e.	p	95% CI	B	t	s.e.	p	95% CI
Muscle density										
Age	−0.081	−12.80	0.006	<0.001	−0.09; −0.07	−0.079	−15.40	0.005	<0.001	−0.09; −0.07
GDF-15	−0.002	−5.03	0.001	<0.001	−0.01; −0.01	−0.002	−3.26	0.001	0.001	−0.01; −0.01
Waist	−0.044	−5.22	0.008	<0.001	−0.06; −0.03	−0.031	−1.72	0.018	0.085	−0.07; −0.01
BMI	−0.078	−4.11	0.019	<0.001	−0.12; −0.04	−0.143	−3.24	0.0,444	<0.001	−0.23; −0.06
Menopause	0.390	2.26	0.172	0.024	0.05; 0.73	–	–	–	–	–
Muscle area										
Age	−11.820	−6.61	1.788	<0.001	−15.32; −8.31	−16.310	−9.69	1.683	<0.001	−19.61; −13.01
GDF-15	−0.338	−2.95	0.114	0.003	−0.56; −0.11	−0.744	−4.82	0.154	<0.001	−1.05; −0.44
Waist	9.667	2.80	3.447	0.005	2.90; 16.43	−13.559	−2.58	5.247	0.010	−23.85; −3.27
BMI	79.411	8.59	9.242	<0.001	61.29; 97.53	183.629	14.11	13.010	<0.001	158.11; 209.15
Menopause	−143.126	−2.70	52.974	0.007	−247.00; −39.25	–	–	–	–	–
Fat area										
Age	13.981	−7.21	1.907	<0.001	−17.79; −10.18	−14.473	−13.41	1.080	<0.001	−16.59; −12.35
GDF-15	−0.011	−0.07	−0.130	0.944	−0.31; 0.29	0.129	1.69	0.076	0.091	−0.02; 0.28
Waist	−10.633	−1.80	2.467	0.072	−22.21; 0.09	14.903	5.54	2.689	<0.001	9.63; 20.18
BMI	166.426	9.81	5.741	<0.001	133.16; 199.69	92.408	12.79	7.224	<0.001	78.24–106.58
Menopause	−60.886	−1.10	−1.10	0.272	−175.26; −47.88	–	–	–	–	–

B, beta coefficients; t, t-value; s.e. standard error; 95% CI, 95% confidence interval.

## 4 Discussion

In this study we investigated the association between GDF-15 and musculoskeletal parameters in a large sample of community-dwelling subjects with a wide age range. Beyond its association with low muscle mass, GDF-15 may also reflect impaired muscle quality. Experimental evidence suggests that GDF-15 is released by stressed skeletal muscle and is considered a cytokine induced by mitochondrial dysfunction (7). Elevated circulating GDF-15 levels have been associated with impaired muscle regeneration, reduced protein synthesis, and increased muscle catabolism. Moreover, chronic inflammation and oxidative stress, both recognized drivers of sarcopenia, may stimulate GDF-15 production, further linking this biomarker to age-related muscle decline.

Reduced skeletal muscle mass and low BMD share common pathophysiological pathways and bidirectional interactions (22). These conditions often co-exist in patients, and the term osteosarcopenia has been proposed as the term to describe this emerging syndrome (23). Bone and muscle interact through multiple communication pathways, and deterioration of musculoskeletal health depends on several risk factors including biochemical, lifestyle, and genetics. Hormones contribute to their homeostasis and growth hormone-insulin-like growth factors 1 play a critical role (22). According to the Mechanical Homeostasis Theory, the reduced mechanical stimuli disrupt bone anabolic processes and lead to microarchitectural deterioration (24, 25). Therefore, skeletal muscle may act as the primary transducer of mechanical loading and provide anabolic signals for bone maintenance. Albeit the exact mechanism of this crosstalk is incompletely understood, several signaling molecules have been found, such as pro-inflammatory cytokines, myostatin, and activin (26, 27).

Our study demonstrated that higher circulating GDF-15 levels were independently associated with lower cortical vBMD in women and lower trabecular and total vBMD in men. These findings are consistent with previous reports showing that elevated GDF-15 is associated with impaired bone health in older adults. Indeed, GDF-15 is involved in many age-related processes and systemic inflammation (28) and is upregulated in response to mitochondrial dysfunction and myocellular stress (29). Indeed, GDF-15 levels are very low in healthy individuals and increase markedly with aging, as well as in chronic and acute conditions (30, 31). Our analysis revealed that GDF-15 is negatively correlated with BMD. These findings are in line with the study by Li et al., who measured GDF-15 in 201 postmenopausal women and found a negative association between GDF-15 and BMD assessed by DEXA at the hip and spine levels. Furthermore, they demonstrated a negative correlation with serum bone alkaline phosphatase, but not with cross-linked N-telopeptides of type I collagen, suggesting that GDF-15 may be involved in reduced bone formation (32). *In vitro* studies further support this hypothesis. Indeed, GDF-15 inhibited the differentiation of cultured human bone marrow-derived mesenchymal stem cells in a dose-dependent manner, increased osteoclast maturation, and reduced the activity of alkaline phosphatase (33). Similar results have been obtained in other skeletal sites, as shown in the study by Hong et al. (34) in which GDF-15 levels exerted a negative modulatory effect on BMD at the distal radius. These authors also suggested that the negative effect of GDF-15 on BMD may be mediated through both endocrine and paracrine mechanisms. A racial difference has been suggested by another study, which showed that GDF-15 was associated with neck BMD in non-African Americans but not in African Americans who had lower levels of GDF-15 (35). Analysis performed in patients with thalassemia showed similar results (18). These clinical findings are consistent with proteomic studies. Indeed, a recent meta-analysis on large-scale circulating proteome association study identified 23 proteins associated with incident hip fractures; among these, the aptamer GDF-15 showed the strongest association with this outcome (HR 1.32, 95% confidence interval 1.21–1.45, *p* = 2.5 × 10^−9^) (19). The same study also reported that protein/aptamer targeting growth hormone receptors showed the strongest association with decreased hip fracture risk, thus underlining the importance of hormonal imbalance in osteoporosis genesis. The mechanism underlying the negative association between GDF-15 and BMD remains unclear. Experimental studies suggest that GDF-15 may inhibit osteoblast differentiation and promote osteoclast maturation. Whether these mechanisms explain the associations observed in our population remains unknown. The concomitant association of GDF-15 with both BMD and muscle mass supports the hypothesis that GDF-15 may reflect processes involving the musculoskeletal unit. Rather than acting independently on bone or skeletal muscle, GDF-15 may represent a marker of systemic mechanisms affecting the musculoskeletal unit, including inflammaging, mitochondrial dysfunction, endocrine alterations, and impaired tissue regeneration. This hypothesis is consistent with the emerging view of GDF-15 as a stress-responsive cytokine and mitokine involved in biological aging and age-related tissue dysfunction (7).

Our study also showed that muscle density and muscle area were negatively correlated with GDF-15. This association has been reported by previous studies. Indeed, the study by Nishikawa et al. reported a negative correlation between GDF-15 and skeletal muscle index, hand grip strength, and walking speed in a cohort of 120 patients undergoing cardiovascular surgery (36). Further, the authors showed that sarcopenia index was a determinant of sarcopenia (37). Similar results have been obtained in patients with chronic obstructive pulmonary disease in the study by Deng et al. who reported that GDF-15 levels were higher in this subset of patients than in controls (38). In addition, serum levels of GDF-15 could predict sarcopenia (area under curve = 0.827). In the Copenhagen PROTECT study analysis showed that GDF-15 above the threshold of 1,541 pg/mL increased the risk of sarcopenia (OR 2.3 in crude models and 2.0 after adjusting for age, gender, Charlson comorbidity index, and C-reactive protein) in a cohort of over 1,000 patients aged at least 65 admitted to the medical ward (14). However, another analysis found no association, as reported in the study by Hansen et al. (39). Although median GDF-15 levels were higher in patients with sarcopenia than in those with normal muscle mass, the authors showed that the area under curve was 0.577, thus demonstrating a poor diagnostic accuracy. The association between GDF-15 and reduced muscle mass observed in the present study is consistent with previous reports in patients with cardiovascular disease, chronic obstructive pulmonary disease, and hospitalized older adults. Therefore, our findings do not establish a novel association *per se*. Rather, the novelty of the present study lies in the simultaneous evaluation of bone and muscle using pQCT in a large community-based cohort, allowing us to investigate GDF-15 as a potential biomarker of the musculoskeletal unit rather than of sarcopenia alone. In addition, we also found sex-specific associations between GDF-15 and pQCT parameters, which may be explained by biological differences in musculoskeletal aging and GDF-15 regulation. Differences in body composition, inflammatory status, bone remodeling patterns, and sex hormones may all influence circulating GDF-15 concentrations and their relationship with bone geometry, density, and muscle characteristics. These mechanisms may underlie the differential associations observed across sexes, although further mechanistic studies are needed to confirm these pathways. The different associations observed between women and men may reflect biological differences in musculoskeletal aging. Sex hormones are major determinants of bone remodeling throughout adult life. Following menopause, estrogen deficiency accelerates intracortical remodeling and cortical porosity, resulting in a more pronounced decline in cortical bone integrity, whereas in men the gradual decline in sex steroids is associated with a slower but more sustained loss of both trabecular and cortical bone (40, 41). These physiological differences may partly explain why, after adjustment for potential confounders, GDF-15 remained independently associated with cortical bone density in women, whereas in men significant associations were retained with trabecular density, total vBMD, and cortical area. Differences in body composition and mechanical loading may also contribute to these findings. Men generally have greater muscle mass and higher habitual mechanical loading of the skeleton, factors that influence bone geometry and periosteal apposition throughout life (42). Consequently, GDF-15 may reflect biological processes affecting both bone density and structural adaptation in males. In contrast, the stronger association with cortical density in women may indicate that GDF-15 is more closely linked to mechanisms involved in cortical bone deterioration after menopause. Finally, sex-specific differences in inflammatory and metabolic regulation should also be considered. Women and men differ in circulating inflammatory mediators, adipose tissue distribution, and immune responses (43), all of which may modulate GDF-15 expression and its downstream effects on bone and skeletal muscle. Since GDF-15 is a stress-responsive cytokine induced by inflammation and mitochondrial dysfunction, these sex-dependent biological pathways may contribute to the different associations observed in our study. Nevertheless, these hypotheses remain speculative and require confirmation in dedicated mechanistic and longitudinal studies.

Our findings support the concept that GDF-15 is associated with both skeletal and muscular deterioration, reinforcing the notion of bone and muscle as an integrated functional unit. The exact mechanism by which GDF-15 correlates with musculoskeletal decline still needs to be elucidated, but a role of mitochondrial dysfunction can be hypothesized. Indeed, mitochondrial dysfunction is a key element of senescence and although the mass of mitochondria is increased in senescent cells, these may be less functional, in part due to the decreased mitophagy (44). Thus, mitochondrial dysfunction may alter muscle metabolism and lead to muscle atrophy (45). Notably, such impairment has been found to be associated with elevated levels of GDF-15 (46). Another possible mechanism is the elevation of mammalian target of rapamycin complex 1 (mTORC1) which increases the expression of GDF-15 (47). Evidence from aging mouse models showed that the inhibition of mTORC1 reduced the expression of GDF-15, thus reducing oxidative stress, muscle damage and muscle loss (47). Anorexia could be another contributing factor. Indeed, the frequency of anorexia increased with aging and is due to different factors, such as drugs, hormonal changes, reduced taste, and alteration in gastrointestinal function (48).

Our study has several important strengths. It was conducted in a large community-based cohort of older adults, providing a robust population for evaluating the association between circulating GDF-15 and musculoskeletal parameters. The sex-stratified analyses allowed us to identify sex-specific associations that might have been overlooked in pooled analyses. In addition, the use of peripheral quantitative computed tomography (pQCT) enabled separate assessment of cortical and trabecular volumetric bone mineral density as well as muscle parameters, offering more detailed information than conventional areal bone mineral density measurements. Finally, standardized laboratory procedures and adjustment for major demographic, anthropometric, and clinical confounders strengthen the reliability of the observed associations. However we acknowledged some limitations. First, gold-standard clinical measurements for diagnosing musculoskeletal decline including DXA and functional performance tests (e.g., handgrip strength) were not available within this historical community-dwelling cohort. Consequently, participants could not be classified according to formal European diagnostic criteria proposed by the European Working Group on Sarcopenia in Older People (EWGSOP2) or the International Osteoporosis Foundation (IOF). However, this limitation is partially counterbalanced by the use of pQCT, which uniquely provides volumetric BMD and compartmental tissue-specific geometric parameters (cortical versus trabecular bone) that cannot be captured via planar DXA. Second, because of the epidemiological design of this large population-based study, the regression analyses were primarily intended to identify independent associations between GDF-15 and musculoskeletal parameters. In addition, the cross-sectional design of the study inherently limits our ability to establish causal relationships between serum GDF-15 levels and musculoskeletal decline. Although age was included as a covariate in all multivariable models, age-stratified and interaction analyses were not performed because of the relatively limited sample size. Finally, we performed multiple regression analyses without formal adjustment for multiple comparisons. Because the investigated outcomes represent biologically related measures of bone and muscle obtained from the same imaging modality and the analyses were based on prespecified hypotheses regarding the association between GDF-15 and musculoskeletal parameters, we did not apply formal multiple-comparison correction. Nevertheless, the possibility of type I error cannot be excluded, particularly for associations with borderline statistical significance, and these findings should therefore be interpreted cautiously and confirmed in independent longitudinal studies.

In conclusion, in this study we found that higher GDF-15 were associated with lower BMD and poorer muscle mass in both males and females. These findings support the role of GDF-15 as a potential biomarker of age-related musculoskeletal deterioration and reinforce the concept of bone and skeletal muscle as an integrated functional unit. Although GDF-15 cannot currently be considered a standalone diagnostic biomarker, it may complement established clinical, biochemical, and imaging assessments by identifying individuals at increased risk of musculoskeletal decline before overt osteoporosis or sarcopenia becomes clinically evident. Future longitudinal studies are needed to determine whether GDF-15 has predictive value for incident osteoporosis, sarcopenia, or fractures, and to clarify its potential role in risk stratification and clinical decision-making. Our findings suggest that GDF-15 may represent a biomarker of age-related deterioration of bone and muscle.

## Data Availability

The raw data supporting the conclusions of this article will be made available by the authors, without undue reservation.
