# Supplementary material for: Association of Serum GDF-15 with Bone and Muscle Parameters in Community-Dwelling Individuals
Source: Front Med (Lausanne). 2026 Oct 9;13:1920342. doi: 10.3389/fmed.2026.1920342 (PMC13690514; doi:10.3389/fmed.2026.1920342)
Supplement: Supplementary file 1 [file Table1.docx]

Supplementary Table 1. adjusted R² values and variance inflation factors (VIFs) of the regression models.

|  | Female | | Male | |
| --- | --- | --- | --- | --- |
|  | Adjusted R^2^ | VIF | Adjusted R^2^ | VIF |
| Total bone density 4% | 0.325 | 3.08 | 0.172 | 3.08 |
| Trabecular bone density 4% | 0.233 | 3.08 | 0.167 | 3.08 |
| Cortical bone density 38% | 0.306 | 3.09 | 0.024 | 3.08 |
| Cortical bone area 38% | 0.162 | 3.09 | 0.139 | 3.08 |
| Total bone area 4% | 0.109 | 3.08 | 0.046 | 3.08 |
| Muscle density | 0.360 | 3.10 | 0.405 | 3.14 |
| Muscle area | 0.283 | 3.10 | 0.321 | 3.14 |
| Fat area | 0.432 | 3.1 | 0.467 | 3.14 |
